# Durability of Stretta Radiofrequency Treatment for GERD: Results of an 8-Year Follow-Up

**DOI:** 10.1155/2014/531907

**Published:** 2014-05-18

**Authors:** Luca Dughera, Gianluca Rotondano, Maria De Cento, Paola Cassolino, Fabio Cisarò

**Affiliations:** ^1^Digestive Motility and Endoscopy, Department of Medicine, Città della Salute e della Scienza, Via Genova, 10121 Turin, Italy; ^2^Gastroenterology and Digestive Endoscopy, Ospedale Maresca, Torre del Greco, Via Montedoro, 80059 Naples, Italy; ^3^Anesthesiology, Department of Surgery, Città della Salute e della Scienza, Via Genova, 10121 Turin, Italy; ^4^Emergency Surgery, Department of Emergency, Città della Salute e della Scienza, Via Genova, 10121 Turin, Italy; ^5^Pediatric Gastroenterology, Department of Medicine, Città della Salute e della Scienza, Via Genova, 10121 Turin, Italy

## Abstract

From June 2002 to March 2013 26 patients that underwent Stretta procedure (16 females, 10 males) reached to date an eight-year follow-up. Primary end point of the study was to verify the durability of the procedure at this time. All patients underwent clinical evaluation by upper endoscopy, oesophageal pressure, and pH studies. For each patient 8-year data were compared to those recorded at baseline and at 4 years. There was a significant decrease in both heartburn and GERD HRQL scores at 4 years (*P* = 0.001) and at 8 years (*P* = 0.003) as well as a significant increase of QoL scores at each control time (mental SF-36 and physical SF-36, *P* = 0.001). After 4 and 8 years, 21 patients (80.7%, *P* = 0.0001) and 20 patients (76.9%, *P* = 0.0001) were completely off PPIs. Median LES pressure did not show significant amelioration at 4 and 8 years and mean oesophageal acid exposure significantly improved at 4 years (*P* = 0.001) but returned to baseline values after 8 years. This further follow-up study of ours from four to eight years confirms that RF energy delivery for GERD provides durable improvement in symptoms and in quality of life and reduces antireflux drugs consumption.

## 1. Introduction

Gastroesophageal reflux disease (GERD) is a complex disorder resulting from multiple contributing factors, including acid production, lower oesophageal sphincter (LES) tone and location, and anatomic barriers to reflux created by the angle of His and the diaphragmatic hiatus [1]. The major mechanism explaining reflux is an impaired function of the gastroesophageal junction, due to transient lower oesophageal sphincter relaxations (TLESRs), or permanent in patients with hiatal hernia [2].

GERD treatment depends upon symptom severity and individual patient characteristics and often requires long-term medical therapy or laparoscopic surgery. The main goal of treating GERD is the achievement of a sustained better quality of life; since neither medical nor surgical therapy completely fulfills the ideal criteria of being simple, effective, risk-free, and cheap [3, 4], endoscopic approaches to GERD management were conceived as a bridge between medical management and surgical treatment, thus obviating the cost of long-term PPIs treatment and potential risks of laparoscopic surgery [5]. Some endoscopic techniques, like intraluminal plication or mucosal injection, proved to be effective in randomized controlled trials, but most of them were withdrawn from the market due to economic reasons or due to severe complications. To date, the Stretta procedure (Mederi Therapeutics Inc., Greenwich, CT, USA), which applies thermal radiofrequency energy to the LES, still remains an available technique, with documented effectiveness on patient symptom control, quality of life (QoL), oesophageal acid exposure, and LES pressure [6, 7]. There is little data examining the long-term durability of Stretta, with the longest ones reporting results at 48 months [8–10].

The aim of this study was to assess the durability of Stretta procedure over a longer term; we report our experience in selected patients who have been treated and then followed up for at least 8 years.

## 2. Materials and Methods

This is a single centre study; in our institution from June 2002 to March 2013 86 patients were treated with the Stretta procedure for GERD. This is our further follow-up study from four to eight years: out of the 56 patients that reached the end of 2010, the goal of 48-month follow-up and whose outcomes were previously published [10], 26 patients (16 females, 10 males) reached at the time an eight-year follow-up (range 8.0–9.3, median 8.6). All the selected patients underwent clinical evaluation and accepted to be submitted to upper endoscopy, oesophageal pressure, and pH-metric studies. For each single patient of this little cohort data were compared to that recorded at baseline and at 4 years.

All patients met the following criteria: (1) heartburn or acid regurgitation responsive to daily medication with proton pump inhibitors (PPIs); (2) age >18 years; (3) 24-hour pH study (off medication) showing abnormal oesophageal acid exposure (≥4%) and a DeMeester score of more than 14.7; (4) oesophageal manometry showing both normal peristalsis and sphincter relaxation with LES pressure below 11 mmHg and more than 5 mmHg; (5) at upper endoscopy on medications no evidence or low-grade esophagitis (Los Angeles, grades A-B), no hiatal hernia or not longer than 2 cm, and no Barrett's oesopghaus. Coagulation disorders, previous oesophageal or gastric surgery, relevant cardiovascular and metabolic diseases, cancer, psychiatric disorders, or nutritional behaviour disturbs such as anorexia and bulimia were excluded. Patients showing at manometry significant ineffective oesophageal motility (IEM) associated with GERD were also excluded.

The Stretta procedure was performed according to the technique first described by Triadafilopoulos [11] during a deeply sedated upper endoscopy; the operator confirms the eligibility criteria and measures the position of the squamous-columnar junction (used as the approximate location for the gastroesophageal junction); then the endoscope is withdrawn and the RF delivery catheter is introduced orally over a guide wire. The Stretta catheter consists of an inflatable and flexible balloon—basket with four electrode needle sheaths. The operator inflates the balloon 2 cm proximal to the squamous-columnar junction, deploys the electrode needles (22-gauge, 5.5 mm length), and delivers RF energy for 90 seconds; since 2005 the RF delivery protocol was changed and the highest RF energy was subsequently delivered only for 60 seconds. The needles are then withdrawn, the balloon is deflated, and the catheter is rotated 45 degrees. This process is repeated every 0.5 cm, covering an area of 2 cm above and 1.5 cm below the squamous-columnar junction plus six sets below the cardias, for a total of 22 sets of needle deployments.

All procedures were performed on an outpatient, day-hospital basis; for deep sedation we used propofol (100–300 mg i.v.) and remifentanil (0.5–1 mg/kg/h i.v.), with continued cardiorespiratory monitoring by an anesthetist well trained in assistance to the endoscopic procedures. Recovery mean time after procedure was 4–6 hours and all patients were discharged from the hospital within the same day. The median procedure time (from starting endoscopy to the catheter removal) was 50 minutes (45–70 min). Endoscopy was performed immediately after treatment to evaluate the RF induced lesion placement. All patients continued their current PPIs regimen for 30 days and then discontinued all antacid medications. For symptom recurrence a standard step-up protocol was used, starting with antacids followed by H2-receptor antagonists and PPIs until symptom relief was achieved.

The primary outcomes of the study were GERD related symptoms (heartburn score, by a 6-point Likert scale ranging from no symptoms to incapacitating symptoms), GERD health-related QoL score (HRQL), using a 6-point Likert scale for multiple different symptoms, each ranging from no symptoms to incapacitating symptoms [12], and general quality of life, using the medical outcome 36-item Short-Form Health Survey (SF-36) [13]; GERD HRQL improvement was evaluated as a continuous variable and as a dichotomous variable (responder versus nonresponder). Response was a >50% improvement compared with baseline values, as we have previously described [14]. Secondary outcomes included medication use, LES pressure at oesophageal manometry, and oesophageal acidic exposure at pH-metry. Medication usage assessment was performed with the use of patient diaries, and patients were specifically queried about the use of all acid reflux medications, including PPIs, H2 receptor antagonists, antacids, and promotility agents. Patient's data concerning the clinical goals, oesophageal manometry, and pH-metry were compared to those recorded at baseline and at 4 years. Baseline characteristics of the patients are outlined in Table 1.

Statistical analysis normality was assessed graphically and with the Shapiro-Wilkes test. For normally distributed variables, we reported mean values and performed comparisons with unpaired and paired *t*-tests, as appropriate. For variables without normal distributions, we reported median values and performed comparisons with the Mann-Whitney U test two sample statistic or the Wilcoxon matched pairs signed rank test, as appropriate. Analysis of dichotomous data (e.g., medication use) using the chi-square statistic was used.

## 3. Results

Out of 86 patients treated with Stretta from June 2002 to March 2013, 48 did not reach the date of the objective of eight years of follow-up, five patients were lost to follow-up, and in seven patients the RF treatment lost its efficacy after three years (3 patients), four years (3 patients), and six years (1 patient); these patients started again the use of PPI on a daily basis and five of them successfully underwent laparoscopic antireflux surgery within the next two years.

**Figure 1:**
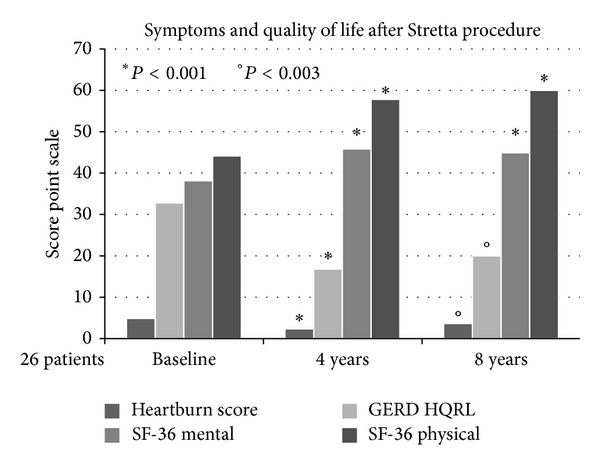
Means report 95% confidence intervals, medians report 25th and 75th percentile ranges, and proportions report percentiles. Heartburn score used a 6-point Likert scale: 0, no symptoms; 1, symptoms noticeable but not bothersome; 2, symptoms noticeable and bothersome, but not every day; 3, symptoms bothersome every day; 4, symptoms that affect daily life; 5, symptoms incapacitating (unable to perform daily activities). For symptom scores the statistical tests compared the mean/median differences in absolute change from baseline values. Heartburn and heartburn-related quality of life (HRQL) score: higher scores for worse symptoms. SF-36 physical score: higher scores for better function.

The Stretta treatment significantly improved heartburn score, GERD HQRL scores, and general QoL scores in all patients at 4 years and at 8 years (Figure 1).

At 4 years there was a significant decrease in the heartburn score (mean decrease, −2.8 points; 95% confidence interval (CI), −1.8 to −3.6; *P* = 0.001) and in GERD HRQL scores (mean decrease, −14 points; 95% CI, −10 to −21; *P* = 0.001). Furthermore, after 8 years this positive effect was still significant both for the heartburn score (mean decrease, −1.8 points; 95% confidence interval (CI), −1.4 to −2.2; *P* = 0.003) and for GERD HRQL scores (mean decrease, −11 points; 95% CI, −9 to −14; *P* = 0.003).

All the patients also showed a very significant increase of general QoL scores at 4 years (mean increase in mental SF-36, 13 points; 95% CI, 8–17; *P* = 0.001 and mean increase in physical SF-36, 12 points; 95% CI, 9–13; *P* = 0.001) and after 8 years (mean increase in mental SF-36, 13 points; 95% CI, 9–15; *P* = 0.001 and mean increase in physical SF-36, 9 points; 95% CI, 6–11; *P* = 0.001).

**Figure 2:**
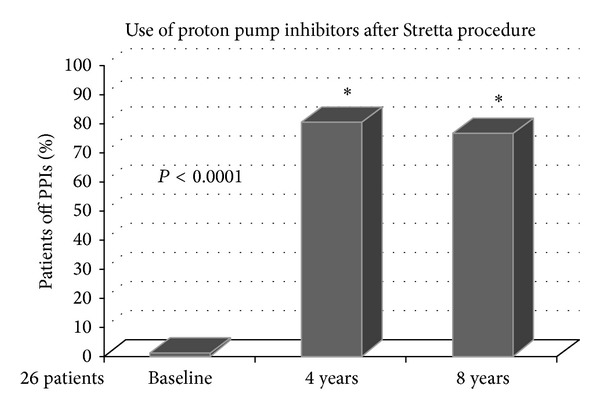
The Stretta procedure significantly reduces the use of antireflux medication. After 4 and 8 years, 21 patients (80.7%, *P* = 0.0001) and 20 patients (76.9%, *P* = 0.0001) were completely off PPIs (including OTC-PPIs and anti-H2 antagonists).

After 4 and 8 years, 21 (80.7%, *P* = 0.0001) and 20 (76.9%, *P* = 0.0001) out of 26 patients were completely off PPIs (including OTC-PPIs and anti-H2 antagonists), whereas some of the others were using occasionally oral antacids or PPIs, none of them on a weekly basis (Figure 2). All the patients after 8 years declared that procedure satisfaction for symptom control was superior to that achieved with prior drug therapy.

The median LES pressure did not show significant amelioration compared to baseline values both at 4 and 8 years. Mean oesophageal acid exposure improved at 4 years (*P* = 0.001) but returned to baseline values after 8 years.

At the endoscopic follow-up at 4 and 8 years none of the treated patients showed esophagitis and did not develop Barrett's oesopghaus or oesophageal cancer. Among the treated patients there were no postprocedure perforations, mucosal lacerations, or bleeding episodes requiring transfusion. The only major adverse event that we recorded was a transient severe gastric paresis in a 52-year-old male patient. The patient had to be hospitalized for 3 weeks and treated with prokinetics, prostigmine, and enteral nutrition, with complete recovery within 8 weeks.

## 4. Discussion

This further follow-up study of ours from four to eight years in 26 consecutive patients submitted to the Stretta procedure evaluated symptom scores, GERD HQRL scores, and general measures of QoL. The oesophageal sphincter pressure and acidic exposure before and after treatment were also measured.

Although the cohort of patients that reached by the end of March 2013 the established follow up is limited, this study supports the concept that the Stretta procedure is durable after 8 years and more, thus obviating the dearth of long-term follow-up data; in fact, at date, the longest studies report data of follow-up not superior to 48 months [11–13]. The RF treatment significantly improved heartburn scores, GERD HRQL scores, and general QoL scores in all patients after 8 years; these results are comparable to what was recorded in shorter term follow-up patient cohorts with similar study design [15]. The procedure satisfaction superior to that achieved with prior drug therapy, although acidic exposure time was not significantly superior in this study to baseline values after 8 years, might be due (1) to the little cohort of data evaluable for this time of follow-up and (2) mostly to the fact that RF delivery might improve reflux and dyspepsia symptoms related to delayed gastric emptying [16].

These results demonstrate that Stretta is an effective option in selected patients with moderate GERD: subjects suffering from heartburn or regurgitation, patients who have adequate oesophageal peristalsis, patients who show 24-hour pH monitoring demonstrating pathologic acid reflux, and individuals who have nonerosive reflux disease or grade A or B esophagitis; moreover, Stretta could fit well for patients who complain of unsatisfactory GERD control with PPIs therapy or have side effects related to PPIs.

At the end of this study 76.9% of patients were completely off PPIs, although the mean basal LES pressure and the mean acidic oesophageal exposure did not show at 8 years significant amelioration compared to pretreatment values. The meta-analysis by Perry and colleagues, covering 18 studies over a 10-year span and including 1441 patients, reported, together with a consistent better symptom control, that oesophageal acid exposure may ameliorate from preoperative values, but pH did not always normalize after Stretta [17]. It was previously established that Stretta procedure significantly reduces TLESRs, the most common underlying cause of GERD, without detrimental effects on peristalsis or swallow-induced LES relaxation pressure [18, 19]. In a more recent double-blind and sham-controlled study the primary outcome was the barostat distensibility test of the gastroesophageal junction before and after administration of sildenafil. The gastroesophageal junction compliance was not significantly altered after the sham procedure, but the active Stretta procedure was associated with a significant decrease in tissue compliance which was normalized after administration of sildenafil. These results indicate that the reduction in tissue compliance may be an important factor and appropriate therapeutic target in the treatment of GERD and that fibrosis is not in fact the cause of this reduction [20]. Neural modulation with impairment of visceral sensory pathways has been extensively debated in the mechanism of action of RF delivery and literature data allow excluding that oesophageal desensitization could be harmful in the natural history of the RF treated patient [21].

The results of this study confirm that Stretta is a safe procedure: in this cohort of patients both at 4 and 8 years we found neither endoscopic esophagitis nor Barrett's oesopghaus nor long-lasting procedure related complications. The meta-analysis by Perry and colleagues showed that the most common complications encountered after the Stretta procedure were transient gastroparesis and erosive esophagitis [17]. Very early reports of oesophageal perforations were attributed to operators' inexperience, and no such severe complications have been reported since then [22].

The results of our further follow-up study from four to eight years sustain the concept that Stretta might represent a viable treatment option for selected patients with symptomatic mild to moderate GERD and this suggestion has been recently stated by the SAGES Guidelines [23]. These results clearly need to be confirmed in larger cohorts of patients, but, if so, it will be reasonable to recommend Stretta to the younger GERD sufferers as a “bridge therapy” between the continuous medical treatment and the optimal timing for laparoscopic fundoplication.

## Figures and Tables

**Table 1:** Baseline characteristics of patients who reached an 8-year follow-up after Stretta procedure (*n* = 26)^a^.

Mean age (yr)	36 ± 18
Male sex	*n* = 10(38.4%)
Mean heartburn score^b^	3.8 ± 1.9
Mean HRQL score^b^	31 ± 7
Mean SF-36 mental^c^	42 ± 9
Mean SF-36 physical^c^	40 ± 6
Daily PPI use	*n* = 26 (100%)
Median 24 hs pH^d^	16.85 (9.9–18.4)
Median LES pressure (mmHg)	9.30 (7.2–11.0)
<2 cm hiatal hernia	*n* = 6 (23%)
Erosive esophagitis at EGD^e^	*n* = 7 (27)

EGD: esophagogastroduodenoscopy; LES: lower oesophageal sphincter.

^a^Means report ± 1 SD, medians report 25th and 75th percentile ranges, and proportions report absolute numbers (percentiles).

^b^Heartburn and heartburn-related quality of life scores (higher scores for worse symptoms, [13]).

^c^SF-36 physical score (higher scores for better function; US general “healthy group” population mean = 55.3 [14]).

^d^Percentage of time of oesophageal pH < 4 (off antisecretory medications).

^e^Six patients with grade A and one patient with grade B esophagitis according to the Los Angeles classification.
